# Estimating Thoracic Movement with High-Sampling Rate THz Technology

**DOI:** 10.3390/s23115233

**Published:** 2023-05-31

**Authors:** Christoph Hoog Antink, Romina Schulz, Maurice Rohr, Konstantin Wenzel, Lars Liebermeister, Robert Kohlhaas, Sascha Preu

**Affiliations:** 1 KIS*MED (AI Systems in Medicine Lab), Technische Universität Darmstadt, 64283 Darmstadt, Germany; 2 Terahertz Devices and Systems, Technische Universität Darmstadt, 64283 Darmstadt, Germany; 3 Fraunhofer Institute for Telecommunications, Heinrich Hertz Institute, 10587 Berlin, Germany

**Keywords:** unobtrusive sensing, terahertz, photonic, respiratory rate

## Abstract

We use a high-sampling rate terahertz (THz) homodyne spectroscopy system to estimate thoracic movement from healthy subjects performing breathing at different frequencies. The THz system provides both the amplitude and phase of the THz wave. From the raw phase information, a motion signal is estimated. An electrocardiogram (ECG) signal is recorded with a polar chest strap to obtain ECG-derived respiration information. While the ECG showed sub-optimal performance for the purpose and only provided usable information for some subjects, the signal derived from the THz system showed good agreement with the measurement protocol. Over all the subjects, a root mean square estimation error of 1.40 BPM is obtained.

## 1. Introduction

Within the last two decades, terahertz (100 GHz–10 THz) technology has undergone strong advancements in terms of emitted THz power of sources as well as low-noise receiver technology. Nowadays, electronic systems offer a dynamic range of up to 120–130 dB at the lower end of the THz range [1]. Photonic systems yet reach more than 110 dB [2,3,4]. Systems are now becoming mature for industrial, bio-technical, and medical applications. Some field examples include the monitoring of car paint thickness and its drying status [5,6,7], monitoring of distribution of pharmaceutics in pills, and skin cancer diagnosis [8]. Terahertz systems offer new approaches to industrially and medically relevant problems due to their unique properties: in contrast to infrared and visible light, THz waves can penetrate through clothing, most common plastics, and paper/envelopes. This enables applications in security-related imaging [9] as well as the aforementioned examples of quality control in industry or monitoring purposes in medicine. While X-rays would also be applicable to many of these problems, they are—in contrast to terahertz waves—ionizing and therefore harmful to human beings and animals. Ultrasound is frequently employed as a substitute for X-rays where possible. However, ultrasound systems face severe challenges with air inclusions in solids that cause high reflections. An example where terahertz outperforms ultrasound is the inspection and quality control of multi-walled tubings [10]. Contactless investigations of such samples under test with ultrasound are therefore scarce and often limited to air-only measurements [11].

In general, several methods to perform the sensing of vital signs from a distance exist [12], ranging from cameras in the visual range over laser-based systems to the use of radar, with early works dating back to the 1970s [13]. Recent microwave concepts [14] have proven to be able to resolve the breathing motion of patients using a six-port radar. In this manuscript, we demonstrate a setup operating at approximately 30 times shorter wavelengths. On principle, this should allow for finer motion resolution while also penetrating through common clothing, paper and plastic. For far-field imaging applications, the resolution of THz systems is typically much better due to the (much) shorter wavelength. The wavelength also affects the resolution along the propagation direction. With an interference-based approach, we have recently achieved a standard deviation of only 31 nm by recording the transmission of wavelengths between 0.5 and 0.375 mm (corresponding to a frequency sweep from 600 to 800 GHz) [15]. The image formation, however, was impeded by the slow speed of the system, requiring ≈24 s per pixel for the frequency sweep, limiting application examples to laboratory use cases only. In this paper, we employ a similar interferometric approach using a much faster THz system with about 45 frequency sweeps per second [16] in order to monitor the chest motion of nine subjects to determine the breathing rate. Clothing was uncontrolled, i.e., subjects were wearing their usual indoor clothing (T-shirt, bra, undergarments).

Abnormal respiratory rates are an indicator of higher mortality among adult patients [17]. Thus, the monitoring of respiration may reveal important information. In particular, unobtrusive respiration monitoring techniques may be beneficial and lead to more accurate predictions, as patients’ awareness of respiration measurement influences the way in which they breath [18]. Especially since the beginning of the COVID pandemic, the interest in remote respiration monitoring techniques has increased [19,20,21], as patient contact needed to be avoided. Contact-based methods in general may cause skin irritation and generate discomfort or soreness in the patient [22], which is especially important for sleep studies or the monitoring of neonates.

## 2. Materials and Methods

### 2.1. THz System

The employed “T-sweeper” system [16] is a photonic continuous-wave terahertz spectrometer that allows up to 200 sweeps per second at a bandwidth of up to 4 THz. The spectrometer consists of a static laser and a swiftly tunable laser (Finisar Wavesource modulated grating Y-branch laser) with a tuning speed of up to 500 THz/s, a repeatability of 50 MHz, and well-known, deterministic mode hops. The fast tunable laser (with a frequency $f_{\mathrm{tun}}$) is combined with the static laser (with a frequency $f_{\mathrm{fix}}$) using a fiber-based 3 dB coupler. The beat note in one port illuminates a pin-diode-based photomixer that converts the beat note to a THz output wave that propagates through the measurement setup and is received by a photoconductor. The other port of the 3 dB coupler is attached to a photoconductive receiver that mixes the THz signal with the optical beat note. The optical distance from the 3 dB coupler via the source and the THz path to the receiver is deliberately chosen to be slightly different from the optical distance from the second port of the 3 dB coupler to the receiver. Due to the fast sweep of the swept laser, the path length difference transforms into a slight frequency offset between the THz signal and the laser signal at the receiver. The rectified THz signal appears thus at an offset frequency (IF). Both the amplitude and phase of the IF signal are recorded and evaluated. The system can be interpreted as a heterodyne spectrometer or likewise a frequency-modulated continuous-wave (FMCW) LIDAR, where the distance is the optical path length difference between the source and receiver path. Further details on the system can be found elsewhere [16]. The setup used in this paper was limited by the maximum data rate and speed of the computer to ≈45 sweeps/s for a scan window from 185 GHz via DC ($f_{\mathrm{tun}}<f_{\mathrm{fix}}$) to 1.314 THz ($f_{\mathrm{tun}}>f_{\mathrm{fix}}$), containing 1500 frequency points. The system is set up in reflection geometry with about $45^{\circ }$ between the source and receiver path. The reflecting object is the chest of the subjects, approximately targeting the sternum. When the sternum moves by Δl, e.g., due to breathing motion, the path length in the THz path changes. This causes a change of the phase as

(1) $$\Delta \varphi =k_{\mathrm{THz}}\Delta l=2\pi /c_{0}f\Delta l.$$

We extract the frequency-dependent phase in order to track the motion of the sternum.

### 2.2. Measurement Protocol

In the measurement protocol, subjects wearing ordinary clothing (T-shirt, pullover, etc.) were asked to breathe normally for 3 min in a sitting position. During breathing, subjects were asked to keep their body in the focus of the THz beam, which was indicated by a time-varying phase signal as well as peaks in the amplitude signal in the live view of the THz system. After this baseline measurement, subjects were ask to perform the following protocol:

- Breath hold for 20 s (or until uncomfortable);
- Controlled breathing at 9 breaths per minute for 30 s;
- Controlled breathing at 12 breaths per minute for 30 s;
- Controlled breathing at 18 breaths per minute for 30 s;
- Resume normal breathing.

The correct breathing rate was ensured by an audible stimulus from a metronome app as well as vocal instructions (“breathe in”, “breathe out”, “hold your breath”, “breathe normally”). After that, subjects were asked to perform light physical exercise and return for another breath hold/normal breathing measurement. In this work, only the control breathing data are used.

For reference data collection, a Polar H10 chest strap was used synchronously. The Polar H10 chest strap allows the recording of raw electrocardiography (ECG) data, which is modulated by respiratory information. The strap is worn below the focal point of the THz beam which ensures no interference with the THz measurement. Thus, a surrogate for a respiratory signal can be obtained from the ECG, which is a process called ECG-derived respiration (EDR) [23]; please see more details below.

A total of nine subjects participated in the measurements, but two recordings had to be discarded because of interruptions in the data acquisition process. All subjects gave informed consent, and the study was approved by the local ethics committee of Technische Universität Darmstadt (EK 13/2022).

### 2.3. THz Signal Processing

**Figure 1:**
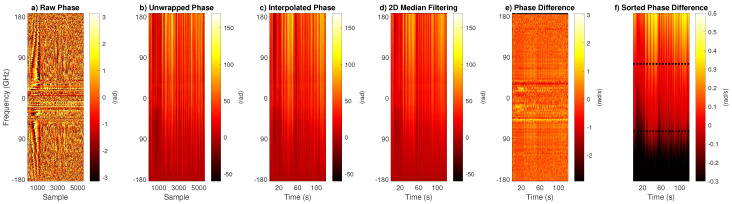
Processing steps of the THz phase signal. In plot (**f**), the color map is arbitrarily clipped at −0.3 and 0.6 for better visualization.

The swept laser is tuned from $f_{\mathrm{fix}}-f_{\mathrm{tun}}=0.185$ THz to $f_{\mathrm{tun}}-f_{\mathrm{fix}}=1.34$ THz with a frequency resolution of 1 GHz. The data contain the frequency interval from DC to 0.185 THz twice as the difference frequency of the two lasers is swept through zero. As also the data quality in this frequency interval was best, we only used this range, which corresponds to the first 372 data points of the phase signal; i.e., information for frequencies beyond 0.185 THz were discarded. In terms of temporal resolution, the system outputs one sweep approximately every 22 ms (standard deviation 8.8 ms, excluding pauses greater than 1 s) and provides a timestamp after data processing. These deviations are created by the limited processing speed of the computer, and the timestamp provided corresponds to the time when the measurement is saved, i.e., after the data are processed by the computer and not the time the measurement is acquired. Figure 1a shows the raw phase information which lies in the range from −π to π, which is subsequently unwrapped (Figure 1b). Next, the unwrapped phase information is interpolated to a regular grid with a sampling time of 10 ms, which corresponds to a sampling rate of 100 Hz (Figure 1c). To remove jumps in the signal, a two-dimensional median filter is deployed. The filter has a size of 4 by 20, where 4 corresponds to the phase-domain (i.e., 4 GHz), and 20 corresponds to the time-domain (i.e., 200 ms). The filtered signal is presented in Figure 1d. To obtain the rate of change of the phase, differences of subsequent phases are calculated as Figure 1e illustrates. The differentiation operation is sensitive to outliers. This becomes obvious when we compare Figure 1e (where the range of the color map is set from −π to π) and Figure 1f. In Figure 1f, the phase differences were sorted by value. As we can see from the figure, the majority of values are covered in this range while only a relatively small fraction exceeds these boundaries. Thus, to obtain the average phase difference signal in a robust way, we used a trimmed mean approach. The lowest $p_{\mathrm{th}}$% of values and the highest $p_{\mathrm{th}}$% of values are discarded before calculating the mean. Here, we set $p_{\mathrm{th}}$ to 30%; i.e., only the 40% values in the middle range are used for calculating the mean, as indicated by the dotted lines in Figure 1f. Note that this does imply that in each time step, the most reliable phase *difference* is used, which does not have to correspond to the same frequency range in each time step.

**Figure 2:**
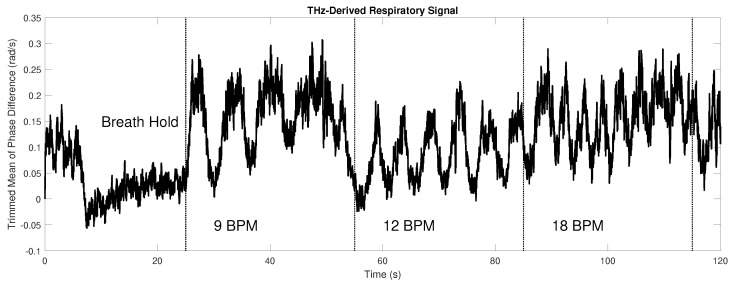
Respiratory signal obtained from the THz phase signal. The oscillation of the phases following the study protocol (breath hold for 20 s, 9 breaths per minute for 30 s, 12 breaths per minute for 30 s, 18 breaths per minute for 30 s) is clearly visible, as each respiratory cycle is represented by a triangular excursion.

Figure 2 shows the resulting phase difference signal. For further processing of the respiratory information, the signal is filtered with a second-order Butterworth filter with a passband of 0.1 to 0.35 Hz.

To obtain the respiratory frequencies from the THz signal, the synchrosqueezed wavelet transform [24] is used. In essence, it comprises a combination of wavelet analysis and a reallocation method. This time-frequency method allows the extraction of instantaneous amplitudes and frequencies, which has proven useful in the extraction of respiratory rate and heart rate, i.e., the instantaneous frequencies of the cardiorespiratory system. From the transformed signal, the maximum absolute value is used to determine the respiratory frequency. The time course of maximum frequencies is finally filtered with a moving median filter with a length of 1401 samples (1.4 s). Note that the median filter is a nonlinear operation that allows the removal of outliers while preserving jumps in the signal, i.e., when the respiratory rate changes.

### 2.4. ECG Signal Processing

**Figure 3:**
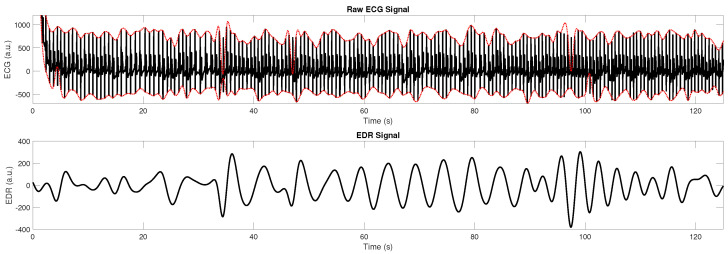
(**Top row**) raw ECG signal with upper and lower envelope (dotted red lines). (**Bottom row**) EDR signal.

The raw information from the ECG chest strap was used to calculate an ECG-derived respiration (EDR) signal. The EDR signal was used for qualitative evaluation of the respiratory signal. For quantitative evaluation, the active part of the study protocol (9 breaths per minute for 30 s, 12 breaths per minute for 30 s, 18 breaths per minute for 30 s) was used, which was automatically identified from the extracted data. Several methods exist to derive EDR. In this work, a straightforward approach that exploits the modulation of the QRS peaks with respiration is used. The QRS complex is the sharp peak in the ECG signal that corresponds to the electric stimulation of the main chambers of the heart before contraction. First, the ECG sample is re-sampled to a fixed sampling rate of 100 Hz using linear interpolation as the raw ECG data are sampled at a mean sampling time of 7.7 ms with a standard deviation of 4.8 ms. Next, a peak-based envelope signal is calculated using MATLAB’s “envelope” function. Here, the envelopes are determined using spline interpolation over local maxima/minima, which were separated by at least 60 samples (corresponding to 600 ms). Next, the lower envelope is subtracted from the upper envelope, and the resulting signal is filtered with a second-order Butterworth filter with cut-off frequencies of 0.1 to 0.35 Hz. An example is given in Figure 3.

## 3. Results and Discussion

**Figure 4:**
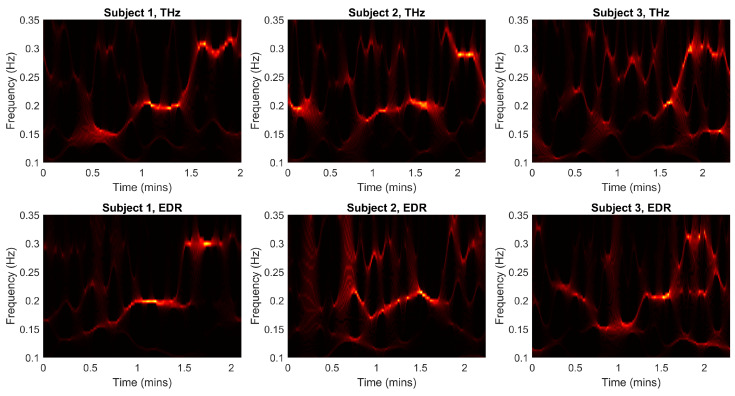

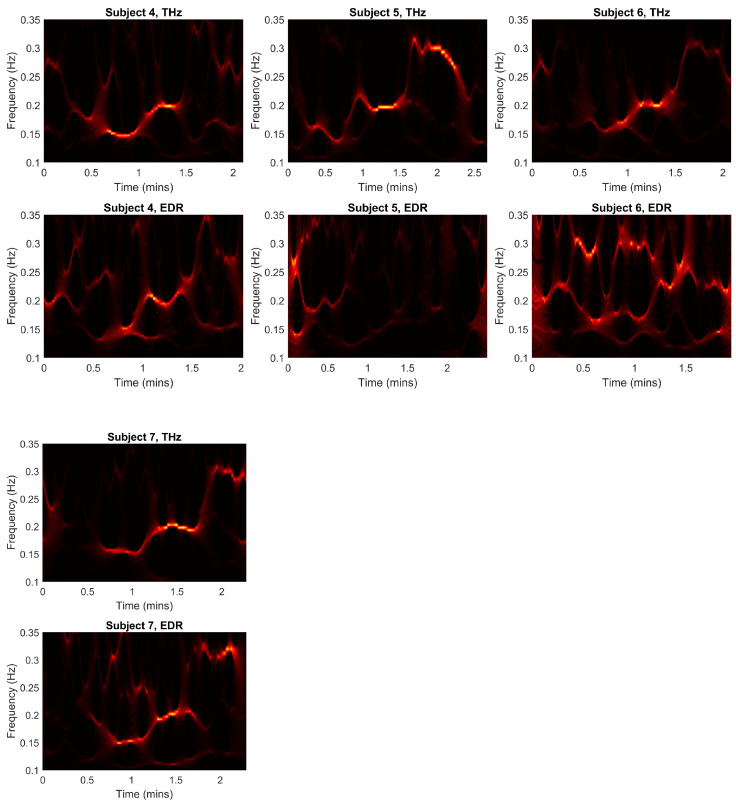
Top rows: Synchrosqueezed wavelet transform of the THz signal. Bottom rows: Synchrosqueezed wavelet transform of the EDR signal.

Figure 4 shows the wavelet synchrosqueezed, filtered transform of the THz phase (first row) as well as the EDR signal (second row) as a qualitative heatmap.

Several observations can be made. First, a good visual agreement of THz and EDR can be determined for subjects 1 and 2. In addition, ridges at the expected frequencies (0.15 Hz/9 BPM, 0.2 Hz/12 BPM, 0.3 Hz/18 BPM) can partly be observed. At the same time, strong variations between subjects can be made out both in the THz as well as the EDR data. Unfortunately, while the ridges show similar behavior, the EDR signal does not show a clear step-like behavior as necessary to qualify as gold standard respiratory reference. In particular for very low respiratory rates, the EDR signal shows very little amplitude, as can be seen by the dark parts in the second row of Figure 4. Thus, for further analysis, the study protocol is used as reference as described above.

**Figure 5:**
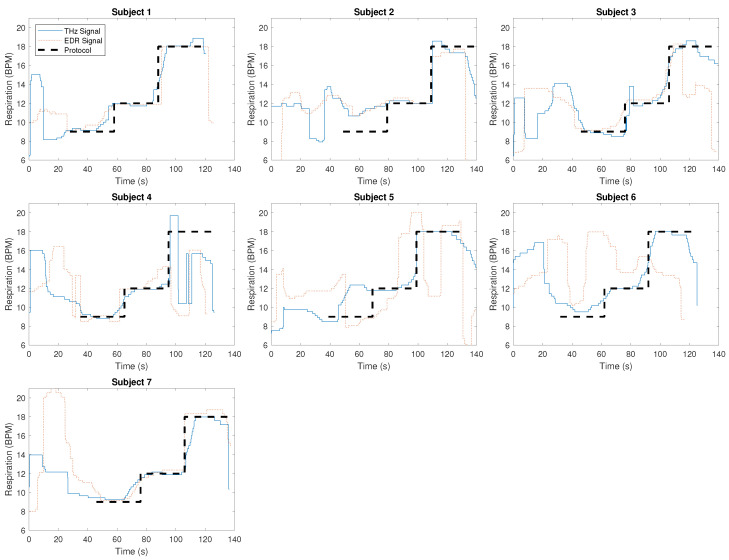
Respiratory rate as extracted from the THz signal (solid blue line), from EDR (dotted red lines), as well as the time-shifted study protocol (dashed black line).

Figure 5 shows the respiratory rate as extracted from the THz signal, the same information extracted from EDR, and the time-shifted study protocol as described above for all seven subjects.

First, the difference between the dotted red line (EDR signal) and the dashed black line (study protocol) further confirms the qualitative observation made from Figure 4, i.e., the inferior quality of the EDR signal. From the overlap of the solid blue line (THz signal) and the dashed black line (study protocol), we can infer that the respiratory rate can be extracted with great accuracy for several subjects (see for example subjects 1, 3, and 7) from the THz signal. At the same time, for some subjects, the respiratory rate estimation shows severe outliers (subject 4). This becomes also obvious from Table 1: While subject 3 shows a very low root mean square error of 0.90 BPM, the error for subject 4 is 2.67 BPM. Over all subjects and respiratory rates, the mean RMSE is 1.40 BPM with a standard deviation of 0.63 BPM.

Note that the precise start of the study protocol was not recorded, and hence, its location was determined based on the data by shifting the study protocol (dashed black line in Figure 5) so as to minimize the error. Thus, the presented results may slightly overestimate the quality of our approach. At the same time, in many cases, the EDR reference shows very good agreement with the last part of the measurement (18 breaths per minute for 30 s), which indicates that fitting the start of the protocol to the data is not likely to have resulted in gross errors. In fact, if for example the time course of THz and EDR-derived information of subject 2 is analyzed in parallel, we may even speculate that the protocol was not followed precisely by the subject and that we might be underestimating the quality of our results. For a fair comparison, the study protocol was also shifted to minimize the error with the EDR approach (not shown in Figure 5). This resulted in only a minor mismatch for subjects 1, 3, and 7 in the range of 0 to 3.5 s. For subject 6, the largest mismatch of estimated protocol starting time in the range of 13 s was observed. Nevertheless, even when this approach is taken, the error between the protocol and EDR approach is much higher compared to THz with a mean RMSE of 2.58 BPM and a standard deviation of 1.52 BPM over all subjects.

Other works have also analyzed the feasibility of using THz for cardiorespiratory monitoring. For instance, in [25], Petkie et al. also proposed a 228 GHz heterodyne radar and showed the general feasibility of monitoring cardiorespiratory activity in a single subject. However, no quantitative analysis of the results was presented, and the frequency used was significant compared to our approach. More recently, Rong et al. demonstrated “terahertz-wave plethysmography”, in which they detected cardiac activity in four subjects that rested their heads on the lab bench with a mean error of 1.51 BPM and a standard deviation of 1.08 BPM [26].

## 4. Conclusions

We have demonstrated respiratory motion recognition with terahertz waves. The data were taken at the lower end of the THz range where the dynamic range of the implemented continuous-wave THz system is highest. The general methodology can be used over the whole working range of the system if a sufficient dynamic range was available. We remark, however, that the frequency range between ≈100 and ≈300 GHz features the best compromise between resolution and transparency of clothing, bedding linen, etc.; i.e., the investigated frequency range is best suited for medical monitoring. Focusing on respiratory motion, we have opted for directing the THz beam at the sternum where the chest motion features a large amplitude. We remark that the methodology can, on principle, also be used for pulse monitoring. For this purpose, however, the THz beam could be directed on a large, superficial blood vessel, e.g., the carotis, for pulse monitoring. Further system improvement, in particular considering the dynamic range, will enable reaching even higher THz frequencies and will give rise to better signal quality and resolution. For a static laboratory case of measurement of the thickness of a dielectric, we have already demonstrated a resolution in the range of 50 nm [15]. Finally, an optimized measurement setup that will eliminate subject swaying, i.e., recording in a lying position, would also help to fully utilize the high resolution of the THz system, in particular for non-contact estimation of heart rate variability or pulse transit time estimations.

## Figures and Tables

**Table 1:** Sample-wise root mean squared error (RMSE) for all subjects as well as mean and standard deviation (sd).

Subject ID	1	2	3	4	5	6	7	Mean	sd
RMSE (BPM)	0.99	1.68	0.90	2.67	1.51	0.99	1.10	1.40	0.63

## Data Availability

The data are not publicly available due to privacy concerns.

## Abbreviations

The following abbreviations are used in this manuscript:

BPM	Breaths per minute
ECG	Electrocardiogram
EDR	ECG-derived respiration
GHz	Gigahertz
RMSE	Root mean square error
SD	Standard deviation
THz	Terahertz
